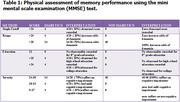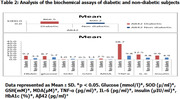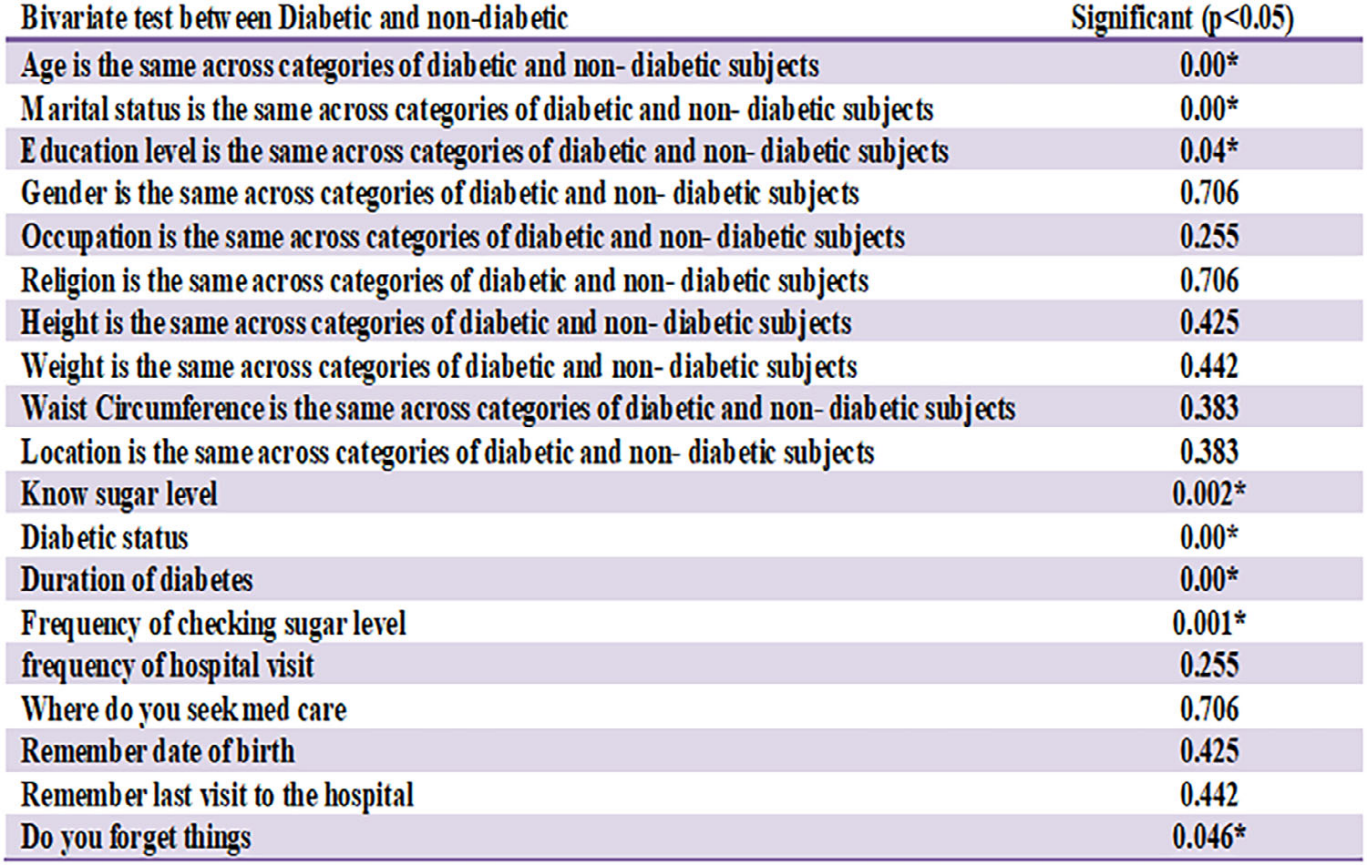# Memory function decline among Nigerian type 2 diabetic patients: Preliminary results from an ongoing multi‐centre case control study

**DOI:** 10.1002/alz.085483

**Published:** 2025-01-09

**Authors:** Juliet Nnenda Olayinka, Emmanuel Irek, Olusegun Adebayo Adeoluwa, Okechukwu Ezekpo, Benjamin Omiyale, Oluwafemi Ogunniyi, Idowu Adarabioyo, Olakunle Aberejo, Lily Otomewo, Oluwaseyi Akpor, Raymond Ozolua

**Affiliations:** ^1^ Afe Babalola University, Ado‐Ekiti Nigeria; ^2^ University of Benin, Benin City Nigeria; ^3^ Afe Babalola University, Ado‐Ekiti, Ekiti State Nigeria; ^4^ Afe Babalola University, Ado Ekiti, Ekiti State Nigeria; ^5^ University of Benin, Benin city, Edo State Nigeria

## Abstract

**Background:**

Type 2 diabetes mellitus (T2DM) is among the modifiable risk factors for Alzheimer's disease (AD) and ranks among the leading chronic diseases globally. It is characterized by elevated blood glucose levels and insulin resistance, which over time may impair memory performance. More so, saliva appears to be a promising biomarker for the diagnosis of AD since conventional methods appear invasive and expensive in the country. Therefore, we investigated the impact of T2DM on memory function in diabetic patients at the Afe Babalola Multisystem Hospital, Nigeria and also determined if saliva may be a reliable diagnostic tool to detect the risk of AD in diabetic patients.

**Method:**

Sixty (60) subjects, consisting of 20 diabetic patients and 40 healthy controls who consented to the study were recruited. A mini‐mental state examination (MMSE) was used to assess the participants’ memory performance. Blood samples were collected from the subjects to estimate biochemical parameters, which include: fasting blood glucose, insulin levels, glycated hemoglobin levels oxidative stress markers (malondialdehyde (MDA), sodium dismutase (SOD), and reduced glutathione (GSH)), as well as inflammatory markers (interleukin‐6 (IL‐6) and tumor necrosis factor‐alpha (TNF‐α)). Saliva was collected to estimate the level of amyloid beta‐42 (Aβ‐42). All these parameters were analyzed using enzyme‐linked immunosorbent assay.

**Result:**

There were significant differences (p<0.05) between the values for glucose, insulin, Aβ‐42, SOD, GSH, MDA, TNF‐α, IL‐6 amongst diabetic and healthy controls. The MMSE result on severity of cognitive impairment showed that 14 (70%) diabetic patients between the scores of 24 and 30 suffer no cognitive impairment, 4 (20%) diabetic patients within the score of 18–23 have mild cognitive impairment, and 2 (10%) diabetic patients between the scores of 0 and 17 suffer severe cognitive impairment in comparison to the healthy control, who suffered no cognitive impairment.

**Conclusion:**

Our results show that T2DM may impair memory performance later in life and that oxidative stress, insulin resistance, elevated levels of Aβ42, increased glycated hemoglobin and inflammation may be implicated in this decline. This ongoing study also strongly suggests that saliva may be an effective and alternative method for the diagnosis of AD in diabetes patients.